# Baseline MRI markers and clinical outcomes during anti-amyloid β therapy: A real-world study in a Japanese dementia center

**DOI:** 10.1016/j.tjpad.2026.100686

**Published:** 2026-10-17

**Authors:** Yuki Kujuro, Marina Saito, Manami Maruko, Kazuko Kamiyama, Aya Nakano, Emi Qian, Teppei Okamura, Daisuke Ito, Toshimi Kageyama, Ichiro Sugiyama, Eriko Ishihara, Tatsuro Kuwahara, Kazunori Moritani, Hitoshi Katai, Hidenori Hattori

**Affiliations:** a Department of Neurology, Tachikawa Hospital, Tachikawa-shi, Tokyo, Japan; b Care Coordination Office, Tachikawa Hospital, Tachikawa-shi, Tokyo, Japan; c Neuropsychology Unit, Tachikawa Hospital, Tachikawa-shi, Tokyo, Japan; d Department of Neurology, Keio University School of Medicine, Shinjuku-ku, Tokyo, Japan; e Department of Radiology, Tachikawa Hospital, Tachikawa-shi, Tokyo, Japan; f Memory Center, Keio University School of Medicine, Shinjuku-ku, Tokyo, Japan; g Department of Cardiology, Tachikawa Hospital, Tachikawa-shi, Tokyo, Japan; h Department of Neurosurgery, Tachikawa Hospital, Tachikawa-shi, Tokyo, Japan; i Department of Psychiatry, Tachikawa Hospital, Tachikawa-shi, Tokyo, Japan; j Department of Gastroenterological Surgery, Tachikawa Hospital, Tachikawa-shi, Tokyo, Japan

**Keywords:** Alzheimer disease, Lecanemab, Donanemab, Medial temporal atrophy, Cerebral microbleeds, Amyloid-related imaging abnormalities

## Abstract

**IMPORTANCE:**

Predictors of decline and of amyloid-related imaging abnormalities (ARIA) during anti-amyloid β therapy in routine care are undefined.

**OBJECTIVE:**

To identify baseline predictors of cognitive, functional, and safety outcomes.

**DESIGN, SETTING, AND PARTICIPANTS:**

Retrospective cohort of 161 consecutive patients initiating anti-amyloid β therapy at a Tokyo dementia center, 2024 to 2026 (lecanemab, 89; donanemab, 72).

**EXPOSURES:**

Lecanemab or donanemab.

**MAIN OUTCOMES AND MEASURES:**

Change in MMSE and CDR-Sum of Boxes (CDR-SB); baseline medial temporal atrophy (VSRAD Z score), amyloid PET burden, and microbleeds; ARIA.

**RESULTS:**

With lecanemab, MMSE and CDR-SB declined significantly at every assessment (CDR-SB +0.79, +1.10, +1.60 at 6, 12, 18 months); memory and orientation declined while language was preserved. Greater baseline medial temporal atrophy was associated with greater 12-month CDR-SB worsening (r = 0.39; *P* = .01; n = 40); patients in the most severe standard category (Z ≥ 3) worsened by 2.62 versus 0.64 points (adjusted β = +2.18; *P* < .001). Atrophy was associated with older age (r = 0.19; *P* = .03) and with baseline memory-related measures — ADAS-Cog score (r = 0.32; *P* < .001) and the MMSE subdomains of delayed recall (β = −0.25; *P* = .006) and time orientation (β = −0.30; *P* = .04) — but not MMSE total score. Amyloid PET burden showed no significant association at most time points. All ARIA events were asymptomatic; ARIA-H occurred in 6 of 25 with microbleeds versus 3 of 136 without (odds ratio, 14.0; *P* < .001). Donanemab follow-up was 6 months.

**CONCLUSIONS AND RELEVANCE:**

Baseline medial temporal atrophy was associated with functional decline and microbleeds with ARIA-H. Pretreatment MRI may provide prognostic and safety information during anti-amyloid β therapy.

## Highlights

• 161 consecutive patients treated with lecanemab or donanemab in routine care.
• Severe medial temporal atrophy predicted 12-month CDR-SB worsening with lecanemab.
• These patients were older and had poorer memory at a similar MMSE total score.
• Any baseline microbleed raised ARIA-H risk 14-fold within eligibility limits.
• Amyloid PET burden appeared to have no association in this cohort.

## 1 Introduction

Anti-amyloid β monoclonal antibodies have entered routine clinical practice, marking a major change in the treatment of early Alzheimer disease. In Japan, lecanemab was launched in December 2023 and donanemab in November 2024, and under the national health insurance system both agents are broadly accessible; approximately 6000 patients received lecanemab in the first year after approval [1]. Randomized trials established that these agents remove cerebral amyloid and slow cognitive decline [2,3], but they provide limited information about which baseline characteristics are associated with subsequent clinical course and treatment-related risk in routine practice.

Two questions now dominate clinical decision-making. The first is prognostic. Treatment effects have been reported almost exclusively as group-level mean changes, yet clinicians must counsel individual patients, and baseline factors associated with subsequent clinical course remain incompletely characterized. Amyloid burden is the biomarker used to establish eligibility, but whether it carries prognostic information once treatment has begun is unknown. The second question concerns safety. Amyloid-related imaging abnormalities (ARIA) are the principal treatment-limiting event, and although trial exclusion criteria and appropriate use recommendations set thresholds for cerebral microbleeds and cortical superficial siderosis [4,5], whether risk is uniform among the patients who meet those thresholds has not been established.

Emerging real-world studies have begun to describe the clinical use of lecanemab, reporting group-level cognitive outcomes and safety from single-center cohorts in Israel, the United Kingdom, the United States, China, and Japan [[6], [7], [8], [9], [10]]. These reports have established feasibility and broad consistency with trial data, but they have largely reproduced the group-level framework of the trials themselves. Real-world cognitive outcome data for donanemab remain scarce, and neither domain-specific trajectories nor individual-level determinants of outcome have been characterized for either agent.

Both questions can in principle be addressed with information that is already generated in routine care. Structural MRI and safety-screening MRI are obtained in every patient before treatment begins, and quantitative measures of medial temporal atrophy, microbleed count, and white matter hyperintensity burden are therefore available at no additional cost or risk to the patient. Whether these routinely acquired markers carry clinically useful information, and how they relate to the amyloid burden on which eligibility is based, has received little attention.

Japan offers an informative setting in which to examine this. Both agents are widely accessible, the treated population is large, and treatment protocols are standardized under national optimal use guidelines. Our hospital is one of the twelve Tokyo Metropolitan Dementia Care Medical Centers and serves as a regional hub for dementia care, and the sequential introduction of lecanemab and donanemab allowed both agents to be observed under identical assessment conditions. In this study we examined cognitive and functional trajectories, domain-level change, treatment persistence, and safety in consecutive patients treated in routine practice, with the principal aim of determining whether baseline MRI markers are associated with subsequent outcome and with ARIA.

## 2 Methods

### 2.1 Participants

This retrospective observational study included consecutive patients who received anti-amyloid β monoclonal antibody therapy between January 2024 and January 2026 at the Dementia Disease Medical Center of Tachikawa Hospital, a regional referral center in Tokyo, Japan. Lecanemab was introduced in January 2024 and donanemab in January 2025. Eligible patients met the Japanese optimal use guidelines for each agent [11,12], including mild cognitive impairment or mild dementia due to Alzheimer disease with MMSE scores of 22–30 for lecanemab or 20–28 for donanemab, a CDR global score of 0.5 or 1, and amyloid positivity on PET or CSF Aβ42/40 analysis. Baseline MRI was performed for safety screening. Under these guidelines, treatment is contraindicated in patients with a history of serious hypersensitivity to the drug, vasogenic edema, five or more cerebral microbleeds, cortical superficial siderosis, or intracerebral hemorrhage exceeding 1 cm; such patients were not treated and are not included. Treatment selection was made by patients after physician explanation and shared decision-making.

### 2.2 MRI monitoring

Brain MRI was performed at baseline and early during therapy according to the Japanese optimal use guidelines [11,12]. For lecanemab, scans were obtained before the 5th, 7th, and 14th infusions; for donanemab, before the 2nd, 3rd, 4th, and 7th infusions. Thereafter, MRI was performed approximately every 6 months. All examinations were performed at our institution on 1.5-T or 3-T systems. Sequences included T1-weighted, T2-weighted, T2*-weighted gradient-recalled echo (GRE), fluid-attenuated inversion recovery (FLAIR), and diffusion-weighted imaging; susceptibility-weighted imaging (SWI) was not used. On the pre-treatment scan, cerebral microbleeds were counted on T2*-weighted GRE images and white matter hyperintensities were graded on FLAIR images using the Fazekas scale [13]. ARIA was identified on scheduled surveillance MRI and classified as ARIA-E, ARIA-H, or both.

### 2.3 Structural MRI analysis

Medial temporal atrophy was quantified with VSRAD advance version 6.00.0006 (Eisai Co, Ltd, Tokyo, Japan), which computes a Z score for a target volume of interest encompassing the entorhinal cortex, hippocampus, and amygdala, referenced to a normal database [14]. Higher Z scores indicate greater atrophy. The software reports the Z score in four categories (0–1, 1–2, 2–3, and 3 or above), and these appear in the routine radiological report for every patient. We used them as reported, and analysed the Z score both continuously and in these four categories; no threshold was chosen from the present data.

### 2.4 Donanemab dosing and premedication

Donanemab dosing regimens evolved over the study period. In the original regimen the dose was 700 mg for the first three infusions and doubled to 1400 mg at the fourth; in the modified regimen the first three doses are titrated stepwise (350, 700, and 1050 mg) before 1400 mg. In the TRAILBLAZER-ALZ 6 study of donanemab, this modified titration reduced the incidence of ARIA-E at 24 weeks by 41% (14% versus 24%) [15], and it was approved in Japan on August 25, 2025. Of 72 donanemab-treated patients, 34 received the original regimen throughout, 6 initiated treatment under the original regimen and were subsequently transitioned to the modified regimen, and 32 received the modified regimen from the outset.

Premedication practice similarly evolved. Of the 72 patients, 15 received no premedication, 6 initiated treatment without premedication and were subsequently transitioned to a clinical pathway incorporating premedication, and 51 received premedication from the first infusion. The premedication regimen consisted of oral acetaminophen (700 mg, 1 hour before infusion) and intravenous dexamethasone (9.9 mg, 30 min before infusion), applied for infusions 1 through 4, and was introduced in June 2025 to reduce the risk of infusion-related reactions. This followed the prescribing information [16] and the approach used in TRAILBLAZER-ALZ 2 [3]. Persistent reactions despite premedication led to treatment discontinuation.

### 2.5 Amyloid confirmation

Amyloid status was verified by PET or CSF Aβ42/40 analysis. CSF assays were used mainly in the early period; after amyloid PET became available at an affiliated hospital it was preferentially used, and nearly all donanemab-treated patients underwent PET. Amyloid PET scans were performed at a single affiliated imaging center, and standardized interpretation reports were provided to ensure consistency.

### 2.6 Cognitive assessment

Certified neuropsychological assessments (MMSE and CDR) were conducted at baseline and approximately every 6 months. To ensure consistency, most evaluations were performed by a single dedicated full-time neuropsychologist, with limited involvement of additional staff during a brief period. ADAS-Cog was performed in a subset of patients when feasible, either as an alternative to CDR or alongside it when time permitted. Because the CDR requires a knowledgeable informant, usually a co-residing family member, it could not be obtained in patients who lived alone or attended unaccompanied; the number of patients contributing to each analysis is therefore reported throughout.

### 2.7 APOE genotyping

APOE genotyping was not performed. It was not reimbursed under the Japanese national health insurance system during the study period; reimbursement became available on July 1, 2026. Testing could be obtained privately, but genetic testing meets cultural reluctance in this setting and few patients requested it after explanation. APOE status was therefore unavailable.

### 2.8 Statistical analysis

Continuous variables are reported as mean (SD) and categorical variables as counts (percentages). Between-group comparisons used the Welch *t*-test or Mann–Whitney U test for continuous variables and the Fisher exact or *χ*² test for categorical variables. Within-group longitudinal change was analysed with paired t tests.

Cross-sectional associations between medial temporal atrophy and baseline amyloid burden, cognitive scores, Fazekas grade, and microbleed count were examined with Pearson correlation. To test whether atrophy was associated with a specific cognitive profile independent of overall severity, each MMSE subdomain score was regressed on the atrophy Z score with adjustment for MMSE total score and age; subdomains with negligible variance (naming, repetition, written command, writing, and copying) were not analysed, and subdomain scores were additionally compared across the four manufacturer-defined atrophy categories (Z score 0–1, 1–2, 2–3, and ≥3), with MMSE total score compared by one-way analysis of variance and trends across categories assessed by Spearman correlation.

Change from baseline was compared between the most severe atrophy category and all others with the Welch *t*-test and by multivariable linear regression adjusted for age, sex, and the corresponding baseline score, and a trend across the four categories was tested with the Spearman rank correlation. Associations between continuous baseline variables and change from baseline were examined with Pearson correlation coefficients, with the number of observations reported for each estimate. Correlations between a baseline score and subsequent change in the same instrument are mathematically coupled and subject to regression to the mean [17]; these are reported but not interpreted as predictors.

Associations between baseline MRI markers and ARIA were assessed with the Fisher exact test and logistic regression. Treatment persistence was assessed by Kaplan-Meier analysis, defining discontinuation as cessation for any cause, including patients who discontinued lecanemab at 18 months; discontinuations at that milestone were excluded from the analysis of reasons for discontinuation. Two-sided *P* < .05 was considered significant. Analyses were performed with Python version 3.11 using the pandas, SciPy, and statsmodels libraries.

### 2.9 Ethical considerations

The study was approved by the Tachikawa Hospital Ethics Committee (approval 2024–09) and complied with the Declaration of Helsinki and Japanese ethical guidelines. The requirement for written informed consent was waived because anonymized data were analysed retrospectively under an opt-out procedure published on the hospital website. All patients provided written informed consent for treatment as part of routine clinical care. This study is reported in accordance with the STROBE statement for observational studies.

## 3 Results

### 3.1 Baseline characteristics

Between January 2024 and January 2026, 89 patients received lecanemab and 72 received donanemab (Table 1). Because the two agents have different eligibility windows under Japanese optimal use guidelines, the two groups differed at baseline in the direction expected from those criteria: MMSE was lower in the donanemab group (23.75 [2.67] vs 24.95 [2.24]; *P* = .003) and CDR-SB was higher (2.73 [1.41] vs 2.17 [0.95]; *P* = .01). Age, sex, ADAS-Cog score, amyloid burden, and medial temporal atrophy did not differ. More than 90% of patients in both groups had a CDR global score of 0.5. The number of patients contributing to each analysis is given throughout. In particular, the CDR requires a knowledgeable informant and could not be obtained in patients who lived alone or attended unaccompanied, and was therefore available in 69 of 89 lecanemab-treated and 53 of 72 donanemab-treated patients.

**Table 1:** Baseline characteristics of the study population.

Variable	Lecanemab	No.	Donanemab	No.	*P* value
Female, No. (%)	61 (68.5)	89	46 (63.9)	72	.650
Age, mean (SD), y	75.7 (8.3)	89	77.8 (7.4)	72	.087
MMSE total, mean (SD)	24.95 (2.24)	87	23.75 (2.67)	72	.003
CDR global score 0.5, No. (%)	75 (93.8)	80	57 (90.5)	63	.679
CDR global score 1.0, No. (%)	5 (6.2)	80	6 (9.5)	63	—
CDR-SB, mean (SD)	2.17 (0.95)	69	2.73 (1.41)	53	.014
ADAS-Cog, mean (SD)	13.00 (4.79)	76	14.33 (4.67)	56	.112
Medial temporal atrophy, VSRAD Z score, mean (SD)	2.02 (0.92)	77	2.06 (0.92)	65	.830
Amyloid PET, Centiloid, mean (SD)	72.8 (21.9)	54	67.6 (26.5)	71	.231
CSF Aβ42/40 ratio, mean (SD)	0.049 (0.009)	34	—	1	—

Data are presented as mean (SD) unless otherwise indicated. The number of patients contributing data is given for each variable, because cognitive and biomarker assessments were not available in all patients under real-world clinical conditions. *P* values were calculated with the Welch *t*-test for continuous variables and the *χ*² test for categorical variables. CSF Aβ42/40 was measured almost exclusively in the lecanemab cohort, before amyloid PET became available at an affiliated hospital, and the single donanemab-treated patient with a CSF measurement is not summarized.

ADAS-Cog, Alzheimer Disease Assessment Scale–Cognitive Subscale; CDR-SB, Clinical Dementia Rating–Sum of Boxes; CSF, cerebrospinal fluid; MMSE, Mini-Mental State Examination; SD, standard deviation; VSRAD, voxel-based specific regional analysis system for Alzheimer's disease.

### 3.2 Cognitive and functional trajectories with lecanemab

In the lecanemab cohort, MMSE declined from 24.95 (2.24) at baseline to 24.00 (2.71) at 6 months, 23.02 (3.02) at 12 months, and 22.62 (2.79) at 18 months. The mean change from baseline was significant at every assessment (−0.80 [2.59] at 6 months, *P* = .01; −1.78 [3.22] at 12 months, *P* < .001; −2.26 [2.68] at 18 months, *P* < .001). CDR-SB rose from 2.17 (0.95) to 3.02 (1.52), 3.38 (1.85), and 3.83 (2.07), with mean increases of +0.79 (1.14), +1.10 (1.52), and +1.60 (1.68) (all *P* < .001). Trajectories are shown in Fig. 1B, D, and full data in Supplementary Table S1. Absolute CDR-SB values were lower than those reported in the phase 2 lecanemab study at both baseline (2.17 vs 2.97) and 18 months (3.83 vs 4.13), consistent with an earlier-stage cohort, while the magnitude of change over 18 months was of similar order (+1.60 vs +1.16).

**Fig. 1:**
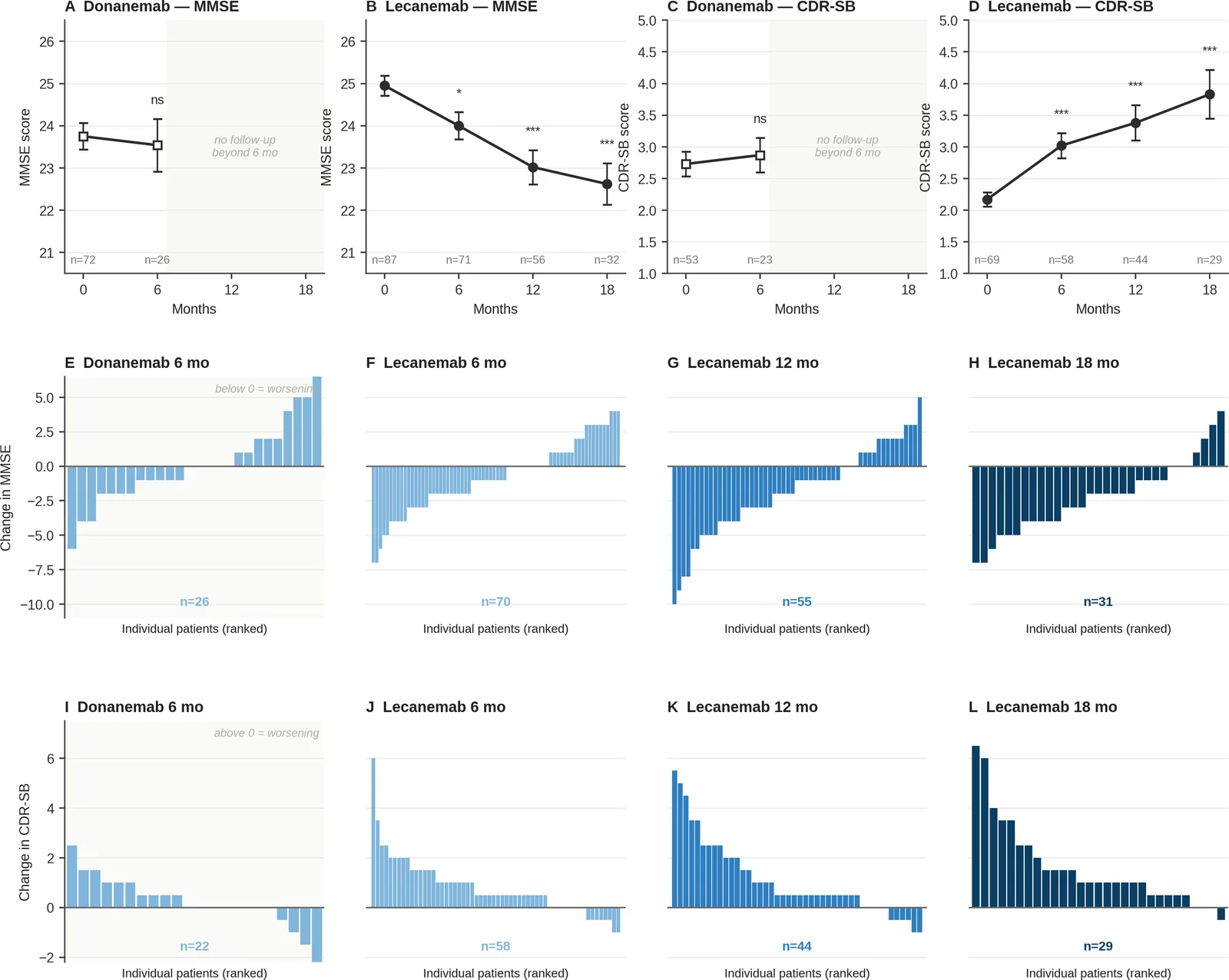
Cognitive and functional trajectories with lecanemab and donanemab. (A–D) Mean scores with standard error at each assessment. Sample sizes are indicated beneath each time point. Asterisks denote paired comparisons with baseline (*P < .05; ***P < .001; ns, not significant). Shading marks the period beyond the available donanemab follow-up. (E–H) Individual change in MMSE and (I–L) in CDR-SB from baseline, with patients ranked by magnitude of change within each panel. The vertical axis is unified within each row. Color indicates time point, not treatment agent. CDR-SB, Clinical Dementia Rating-Sum of Boxes; MMSE, Mini-Mental State Examination; SE, standard error.

### 3.3 Individual variability

Individual trajectories were heterogeneous (Fig. 1F–H, J–L). At 6 months, 38 of 70 patients (54%) had declined on MMSE, 12 (17%) were unchanged, and 20 (29%) had improved; by 18 months these proportions were 24 of 31 (77%), 3 (10%), and 4 (13%). On CDR-SB the proportion worsening rose from 41 of 58 (71%) at 6 months to 25 of 29 (86%) at 18 months. A minority remained stable or improved at every time point.

### 3.4 Domain-level change with lecanemab

Decline was not uniform across cognitive and functional domains (Supplementary Fig. S1). At 6 months, significant worsening was confined to CDR memory, orientation, community affairs, and home and hobbies, together with MMSE orientation to place. By 12 months, MMSE orientation to time and delayed recall had also declined significantly, and CDR personal care was affected. At 18 months, significant worsening involved MMSE orientation to time and place and CDR memory, orientation, community affairs, home and hobbies, and personal care. Naming, repetition, writing, copying, and immediate recall showed no significant change at any time point.

### 3.5 Donanemab

Donanemab was introduced in January 2025, and follow-up was therefore limited to 6 months; these data are presented descriptively and no conclusion about the longer-term trajectory of this cohort can yet be drawn. At 6 months, MMSE was 23.54 (3.18) (mean change +0.08 [2.95]; *P* = .90; n = 26) and CDR-SB was 2.87 (1.30) (mean change +0.23 [1.05]; *P* = .32; n = 22). Trajectories and the distribution of individual change are shown in Fig. 1A, C, E, I, and no MMSE or CDR subdomain changed significantly (Supplementary Fig. S1). Baseline CDR-SB in this cohort (2.73 [1.41]) was substantially lower than in TRAILBLAZER-ALZ 2 overall (3.92 [2.06]) and in its low-to-medium tau subgroup (3.72 [2.09]).

### 3.6 Predictors of cognitive and functional decline

Baseline medial temporal atrophy was associated with subsequent functional decline in the lecanemab cohort, in which follow-up extended to 18 months (Fig. 2A, B). Among patients with 12-month CDR-SB data (n = 40), greater baseline atrophy was associated with greater worsening in CDR-SB when the Z score was analysed continuously (r = 0.39; *P* = .01) (Fig. 2A). When patients were grouped by the four standard VSRAD categories (Z 0–1, 1–2, 2–3, and ≥3), mean change was +0.50, +0.87, +0.42, and +2.62 points, respectively, with the greatest worsening confined to the most severe atrophy category (Fig. 2B). Patients with Z ≥ 3 worsened by 2.62 (2.28) points compared with 0.64 (0.96) points in those with Z < 3 (*P* = .04), and 6 of 8 (75%) worsened by at least 1 point compared with 10 of 32 (31%) (*P* = .04). The association remained significant after adjustment for age, sex, and baseline CDR-SB (β = +2.18; 95% CI, 1.04 to 3.32; *P* < .001).

**Fig. 2:**
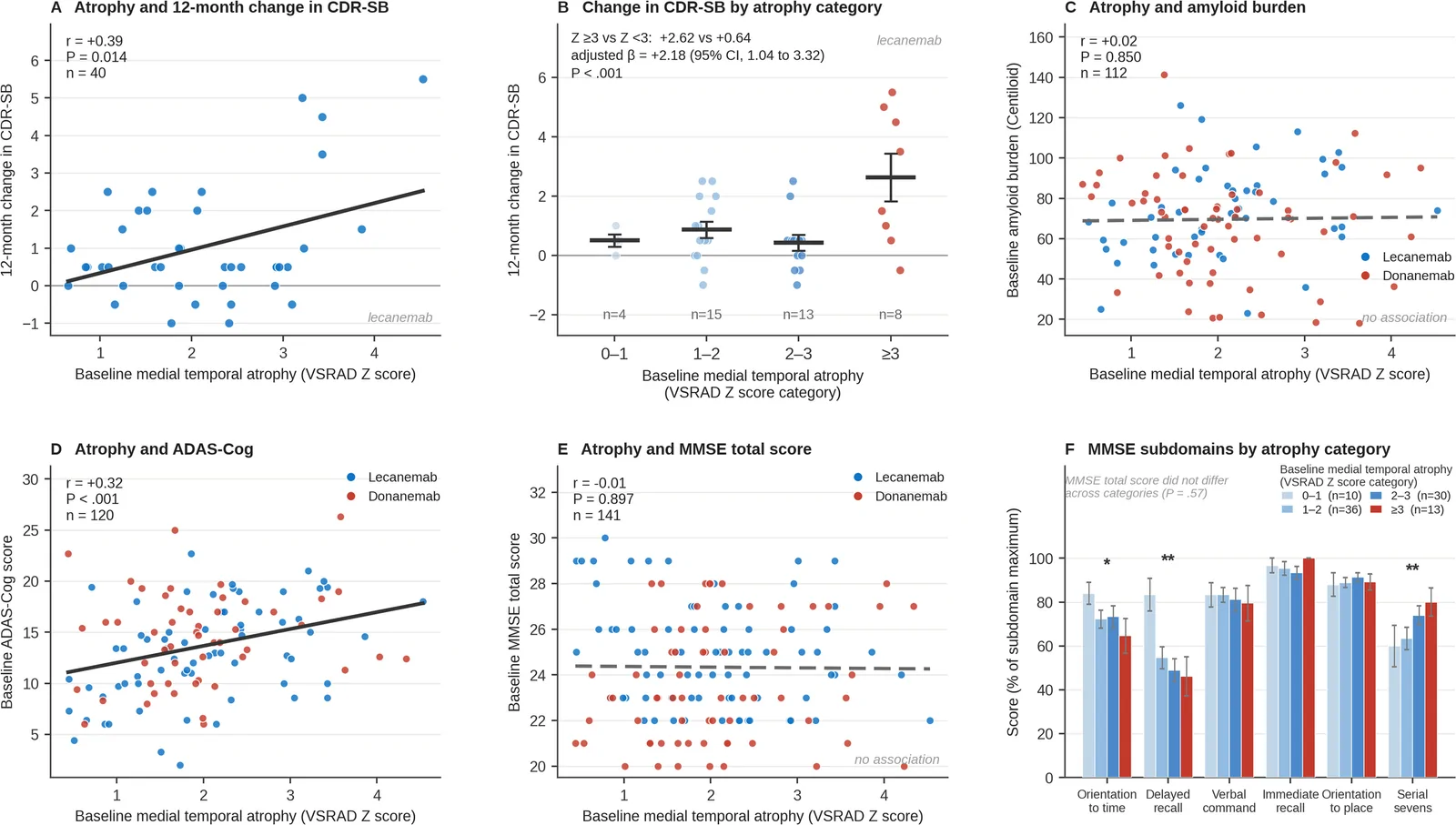
Baseline medial temporal atrophy predicts subsequent functional decline and identifies a baseline memory-predominant cognitive profile. (A) Baseline medial temporal atrophy Z score plotted against change in CDR-SB from baseline to 12 months in lecanemab-treated patients (n = 40). The solid line is the least-squares fit. (B) The same lecanemab data grouped by the four standard VSRAD categories of the baseline Z score. Each point is one patient; horizontal bars indicate the group mean and vertical bars the standard error. Patients in the most severe category (Z ≥ 3) worsened by 2.62 points compared with 0.64 points in those below that threshold, a difference that persisted after adjustment for age, sex, and baseline CDR-SB. Panels (A and B) are confined to the lecanemab cohort, in which 12-month follow-up was available. Panels (C–F) concern the baseline profile of patients with medial temporal atrophy and therefore combine the two cohorts; blue denotes lecanemab-treated and red donanemab-treated patients. (C) Baseline medial temporal atrophy Z score plotted against baseline amyloid burden (n = 112), (D) against baseline ADAS-Cog score (n = 120), and (E) against baseline MMSE total score (n = 141). Solid lines indicate a significant least-squares fit and dashed lines a non-significant fit; r, P, and n are given in each panel. The number of patients differs between panels because not every baseline assessment was available in every patient. (F) MMSE subdomain scores, expressed as a percentage of each subdomain maximum, in the four standard VSRAD categories of the baseline atrophy Z score, in the 89 patients (65 lecanemab, 24 donanemab) with item-level MMSE data. Total MMSE score did not differ across the four categories (25.3, 24.1, 24.5, and 24.5; *P* = .57). Bars show mean and standard error. Asterisks denote the significance of the atrophy Z score in linear regression of each subdomain on atrophy, adjusted for MMSE total score and age (*P < .05; **P < .01). Subdomains with negligible variance (naming, repetition, written command, writing, and copying) were not analysed. Higher Z scores indicate greater atrophy. Positive change in CDR-SB and higher ADAS-Cog scores indicate worse status; higher MMSE scores indicate better status. ADAS-Cog, Alzheimer Disease Assessment Scale–Cognitive Subscale; CDR-SB, Clinical Dementia Rating–Sum of Boxes; CI, confidence interval; MMSE, Mini-Mental State Examination; VSRAD, voxel-based specific regional analysis system for Alzheimer's disease.

A similar pattern was observed for MMSE at 6 months. Mean change was −2.18 (1.66) in patients with Z ≥ 3 and −0.54 (2.76) in those with Z < 3 (*P* = .02; n = 65), with a graded relationship across the four atrophy categories (ρ = −0.29; *P* = .02). After adjustment for age, sex, and baseline MMSE, the estimated difference remained similar in magnitude but was of borderline statistical significance (β = −1.68; 95% CI, −3.36 to 0.01; *P* = .05).

We next examined which baseline characteristics were associated with medial temporal atrophy. Because this analysis concerns the baseline profile of patients with medial temporal atrophy rather than treatment outcome, the two cohorts of both drugs were combined (Fig. 2C–F). Greater atrophy was associated with older age (r = 0.19; *P* = .03; n = 142), whereas it was not associated with baseline amyloid burden (r = 0.02; *P* = .85; n = 112) (Fig. 2C), Fazekas grade (r = −0.09; *P* = .27), or microbleed count (r = 0.00; *P* = .99). We then examined baseline cognitive performance. ADAS-Cog score, which is weighted towards memory, was associated with atrophy (r = 0.32; *P* < .001; n = 120) (Fig. 2D), whereas MMSE total score was not (r = −0.01; *P* = .90; n = 141) (Fig. 2E).

Because MMSE total score was unrelated to atrophy, we examined the MMSE subdomains, which were available in 89 of these patients (65 receiving lecanemab and 24 receiving donanemab). MMSE total score did not differ across the four atrophy categories in this subgroup (25.3, 24.1, 24.5, and 24.5 points for Z scores of 0–1, 1–2, 2–3, and ≥3; *P* = .57), but the pattern of subdomain performance did (Fig. 2F). Delayed recall fell progressively with increasing atrophy (83.3%, 54.6%, 48.9%, and 46.2% of the maximum score; ρ = −0.275; *P* = .009), whereas performance on serial sevens rose (60.0%, 63.3%, 74.0%, and 80.0%; ρ = +0.236; *P* = .026). Orientation to time showed the same downward direction without reaching significance (84.0%, 72.2%, 73.3%, and 64.6%; *P* = .25). After adjustment for MMSE total score and age, greater atrophy was associated with poorer delayed recall (β = −0.25; *P* = .006) and orientation to time (β = −0.30; *P* = .04), and with better performance on serial sevens (β = +0.43; *P* = .003). Immediate recall, orientation to place, and verbal command were not associated with atrophy. Patients with severe medial temporal atrophy, who showed the greatest 12-month worsening in CDR-SB, were therefore already distinguishable at baseline by older age and by a memory-predominant pattern of impairment at an equivalent MMSE total score.

We next examined whether baseline amyloid burden was associated with subsequent change. Baseline Centiloid value was associated with concurrent ADAS-Cog score in the two cohorts combined (r = 0.24; *P* = .02; n = 105), indicating an association with cognitive status at the time of measurement. In the lecanemab cohort, estimates for subsequent change consistently favoured greater decline at higher Centiloid values, but none reached significance at 6 or 12 months (ΔCDR-SB, r = 0.18 and 0.29; ΔMMSE, r = −0.18 and 0.02). An inverse correlation with 18-month MMSE change did reach significance (r = −0.75; *P* = .02), but rested on 9 patients and was not corroborated by 18-month CDR-SB change in the same patients (r = 0.31; *P* = .45). The CSF Aβ42/40 ratio was not associated with change in any outcome (Table 2).

**Table 2:** Baseline variables and subsequent change in cognitive and functional outcomes.

A. Change in MMSE				
Baseline variable	Lecanemab 6 mo	Lecanemab 12 mo	Lecanemab 18 mo	Donanemab 6 mo
Age	−0.03 (.818) [70]	−0.02 (.896) [55]	−0.18 (.336) [31]	−0.18 (.389) [26]
Sex	−0.02 (.902) [70]	−0.07 (.595) [55]	0.27 (.138) [31]	0.24 (.232) [26]
Baseline MMSE	−0.35 (.003)* [70]	−0.41 (.002)* [55]	−0.26 (.161) [31]	−0.38 (.056) [26]
Baseline CDR-SB	−0.18 (.178) [59]	0.04 (.817) [45]	−0.19 (.325) [28]	0.11 (.617) [23]
Baseline ADAS-Cog	−0.11 (.399) [62]	0.06 (.685) [49]	−0.34 (.093) [26]	−0.18 (.383) [25]
Medial temporal atrophy (VSRAD Z)	−0.28 (.022)* [65]	−0.09 (.543) [51]	−0.24 (.224) [27]	−0.24 (.262) [24]
Amyloid PET (Centiloid)	−0.18 (.270) [39]	0.02 (.939) [28]	−0.75 (.019)* [9]	0.12 (.545) [26]
CSF Aβ42/40	0.11 (.556) [32]	−0.07 (.716) [29]	0.07 (.763) [22]	—

B. Change in CDR-SB				
Baseline variable	Lecanemab 6 mo	Lecanemab 12 mo	Lecanemab 18 mo	Donanemab 6 mo
Age	0.03 (.798) [58]	0.04 (.794) [44]	0.07 (.728) [29]	−0.12 (.597) [22]
Sex	0.16 (.231) [58]	−0.11 (.471) [44]	−0.19 (.311) [29]	−0.18 (.421) [22]
Baseline MMSE	−0.12 (.375) [57]	−0.00 (.987) [43]	−0.19 (.324) [28]	0.17 (.446) [22]
Baseline CDR-SB	0.03 (.796) [58]	0.06 (.701) [44]	0.15 (.426) [29]	−0.37 (.090) [22]
Baseline ADAS-Cog	0.03 (.845) [50]	0.27 (.101) [38]	0.42 (.040)* [24]	−0.16 (.486) [21]
Medial temporal atrophy (VSRAD Z)	−0.09 (.511) [53]	0.39 (.014)* [40]	0.13 (.529) [25]	0.21 (.372) [20]
Amyloid PET (Centiloid)	0.18 (.341) [31]	0.29 (.236) [19]	0.31 (.451) [8]	0.17 (.457) [22]
CSF Aβ42/40	−0.28 (.155) [27]	0.28 (.181) [25]	0.24 (.292) [21]	—

C. Change in ADAS-Cog				
Baseline variable	Lecanemab 6 mo	Lecanemab 12 mo	Lecanemab 18 mo	Donanemab 6 mo
Age	−0.23 (.258) [27]	−0.08 (.712) [22]	−0.13 (.758) [8]	−0.53 (.117) [10]
Sex	0.07 (.739) [27]	0.22 (.317) [22]	0.46 (.248) [8]	−0.09 (.812) [10]
Baseline MMSE	0.19 (.342) [27]	0.55 (.009)* [22]	−0.25 (.549) [8]	−0.49 (.147) [10]
Baseline CDR-SB	−0.26 (.310) [17]	−0.10 (.757) [13]	0.34 (.509) [6]	0.55 (.159) [8]
Baseline ADAS-Cog	−0.49 (.009)* [27]	−0.48 (.024)* [22]	−0.18 (.662) [8]	0.45 (.188) [10]
Medial temporal atrophy (VSRAD Z)	−0.02 (.941) [27]	0.01 (.966) [22]	−0.19 (.651) [8]	−0.35 (.355) [9]
Amyloid PET (Centiloid)	0.05 (.848) [17]	0.40 (.160) [14]	—	0.16 (.669) [10]
CSF Aβ42/40	−0.50 (.094) [12]	0.20 (.588) [10]	0.35 (.567) [5]	—

Values are Pearson correlation coefficients (r) with *P* values in parentheses and the number of patients in brackets. Asterisks denote *P* < .05. Sex was coded 1 = male and 2 = female, so a positive coefficient indicates a larger change in women. Correlations between a baseline score and subsequent change in the same instrument are mathematically coupled and subject to regression to the mean, and are not interpreted as predictors.

ADAS-Cog, Alzheimer Disease Assessment Scale–Cognitive Subscale; CDR-SB, Clinical Dementia Rating–Sum of Boxes; CSF, cerebrospinal fluid; MMSE, Mini-Mental State Examination; VSRAD, voxel-based specific regional analysis system for Alzheimer's disease.

Correlations between a baseline score and subsequent change in the same instrument are reported in Table 2 but are mathematically coupled and are therefore not interpreted as predictors.

### 3.7 Safety and treatment discontinuation

All 11 ARIA events were clinically asymptomatic and detected on scheduled surveillance MRI. ARIA-E occurred in 2 of 72 donanemab-treated patients (2.8%), ARIA-E with ARIA-H in 2 (2.8%), and isolated ARIA-H in 2 (2.8%); no ARIA-E occurred with lecanemab, in which ARIA-H occurred in 5 of 89 patients (5.6%), including one case with intracerebral hemorrhage (Table 3A). Individual donanemab ARIA events, with the infusion number, dosing regimen, and premedication status at the time of the event, are listed in Supplementary Table S2.

**Table 3:** Safety, treatment discontinuation, and baseline cerebrovascular markers.

A. Adverse events			
Event	Lecanemab (n = 89), No. (%)	Donanemab (n = 72), No. (%)	
ARIA-E	0	2 (2.8)	
Isolated ARIA-H	4 (4.5)	2 (2.8)	
ARIA-E + ARIA-H	0	2 (2.8)	
ARIA-H with intracerebral hemorrhage	1 (1.1)	0	
Subarachnoid hemorrhage	1 (1.1)	0	
Infusion-related reaction	2 (2.2)	8 (11.1)	

B. Reasons for treatment discontinuation before 18 months			
Reason	Lecanemab (n = 11), No. (%)	Donanemab (n = 8), No. (%)	
Infusion-related reaction	1 (9.1)	4 (50.0)	
ARIA-E + ARIA-H	0	1 (12.5)	
ARIA-H with intracerebral hemorrhage	1 (9.1)	0	
Subarachnoid hemorrhage	1 (9.1)	0	
Trauma	3 (27.3)	0	
Disease progression	2 (18.2)	0	
Patient or family request	1 (9.1)	3 (37.5)	
Other comorbidities	1 (9.1)	0	

C. Baseline cerebrovascular markers and their association with ARIA-H			
	Lecanemab (n = 89)	Donanemab (n = 72)	*P* value
Any cerebral microbleed, No. (%)	12 (13.5)	13 (18.1)	.51
1 microbleed	9 (10.1)	8 (11.1)	
≥2 microbleeds	3 (3.4)	5 (6.9)	
Cortical superficial siderosis, No. (%)	0 (0)	0 (0)	—
Fazekas grade, No. (%)			.31
Grade 0	8 (9.0)	4 (5.6)	
Grade 1	66 (74.2)	52 (72.2)	
Grade 2	12 (13.5)	15 (20.8)	
Grade 3	3 (3.4)	1 (1.4)	
Fazekas grade ≥2, No. (%)	15 (16.9)	16 (22.2)	.43
ARIA-H by baseline microbleed status			
No microbleed	1/77 (1.3)	2/59 (3.4)	
Any microbleed	4/12 (33.3)	2/13 (15.4)	
Both cohorts combined	3/136 (2.2) vs 6/25 (24.0)	OR 14.0 (3.19–50.7)	<.001

All ARIA events were asymptomatic and detected on scheduled MRI surveillance. Discontinuations at the 18-month evaluation milestone in the lecanemab group were excluded from the analysis of reasons for discontinuation. Percentages are calculated within each treatment group. In Table 3C, *P* values were calculated with the Fisher exact test (microbleeds, Fazekas grade ≥2, ARIA-H) and the Mann–Whitney test (distribution of Fazekas grade). The odds ratio for ARIA-H compares patients with and without any baseline microbleed in the two cohorts combined.

ARIA, amyloid-related imaging abnormality; ARIA-E, amyloid-related imaging abnormality with edema or effusion; ARIA-H, amyloid-related imaging abnormality with hemorrhage or hemosiderin; CI, confidence interval; MRI, magnetic resonance imaging; OR, odds ratio.

Infusion-related reactions occurred in 8 of 72 donanemab-treated patients (11.1%) and 2 of 89 lecanemab-treated patients (2.2%). All reactions in the donanemab group were immediate hypersensitivity reactions during or at the end of infusion, and four led to discontinuation. Reactions in the lecanemab group were delayed and mild. Individual reactions in the donanemab group are listed in Supplementary Table S3.

Continuation was high in both groups (Supplementary Fig. S2). At 6 months, 92.9% (95% CI, 87.4 to 98.4) of lecanemab-treated and 87.7% (79.1 to 96.3) of donanemab-treated patients remained on treatment; continuation with lecanemab was 88.9% (82.0 to 95.8) at 12 months and 82.9% (73.6 to 92.2) at 18 months. Most discontinuations occurred early. Among 32 patients reaching the 18-month evaluation on lecanemab, 29 (90.6%) continued beyond that point. Reasons for discontinuation are summarised in Table 3B.

### 3.8 Baseline cerebrovascular markers

We next examined whether baseline cerebrovascular findings on the pretreatment MRI were associated with the development of ARIA. Baseline cerebral microbleeds were present in 12 of 89 lecanemab-treated patients (13.5%) and 13 of 72 donanemab-treated patients (18.1%; *P* = .51), and all counts were within the permitted range of four or fewer. No patient had cortical superficial siderosis, which is a contraindication to treatment. Fazekas grade 2 or higher was present in 15 of 89 lecanemab-treated patients (16.9%) and 16 of 72 donanemab-treated patients (22.2%; *P* = .43), and grade did not differ between the groups (*P* = .31) (Table 3C).

Baseline microbleeds were strongly associated with ARIA-H, which occurred in 6 of 25 patients with any microbleed (24.0%) and in 3 of 136 without (2.2%) (odds ratio, 14.0; 95% CI, 3.19 to 50.7; *P* < .001). A dose-response relationship was apparent across the whole cohort (no microbleed, 3/136 [2.2%]; one microbleed, 3/17 [17.6%]; two or more, 3/8 [37.5%]; *P* < .001). The association persisted after adjustment for age and treatment agent (odds ratio, 13.3; 95% CI, 3.03 to 58.8; *P* < .001) and for Fazekas grade in addition (odds ratio, 13.2; 95% CI, 2.90 to 59.8; *P* < .001). Baseline Centiloid values did not differ between patients who developed ARIA and those who did not (69.7 [27.7] vs 70.2 [24.4]; *P* = .98), and microbleed count was uncorrelated with the Centiloid value (r = 0.03; *P* = .71). The estimate for ARIA-E was in the same direction but imprecise, with only four events.

## 4 Discussion

The principal finding of this real-world study was that routinely available baseline MRI markers were associated with subsequent clinical and safety outcomes during anti-amyloid β therapy, whereas baseline amyloid PET burden did not reach statistical significance at most time points. Among lecanemab-treated patients, severe medial temporal atrophy (VSRAD Z score ≥ 3) was associated with substantially greater worsening in CDR-SB at 12 months, even after adjustment for age, sex, and baseline CDR-SB. Baseline cerebral microbleeds were similarly associated with subsequent ARIA-H, with an apparent increase in risk as microbleed count increased. In contrast, amyloid PET burden was associated with cognitive status at baseline but was not associated with subsequent cognitive or functional change or with ARIA. These findings suggest that the structural and vascular information already available on pretreatment MRI may complement amyloid biomarkers in clinical assessment before anti-amyloid therapy.

The association between medial temporal atrophy and subsequent functional decline is particularly relevant because the atrophy categories used in this study were predefined by the VSRAD software and were not derived from the present data. Patients with a Z score ≥ 3 worsened by a mean of 2.62 points on CDR-SB at 12 months, compared with 0.64 points among those with less severe atrophy, and the association remained significant after adjustment for age, sex, and baseline CDR-SB. A similar direction was observed for MMSE decline at 6 months, with a graded relationship across the four standard atrophy categories. Importantly, baseline atrophy and subsequent change in CDR-SB were measured using different instruments, avoiding the mathematical coupling that complicates interpretation of associations between a baseline score and subsequent change in the same measure [17]. Analysed continuously, the Z score was also associated with 12-month CDR-SB change, and the findings consistently suggest that greater baseline medial temporal neurodegeneration may be associated with a less favorable subsequent clinical course.

Medial temporal atrophy did not simply parallel the other markers of Alzheimer disease severity available in this cohort. It was unrelated to amyloid PET burden, white matter hyperintensity burden, microbleed count, or MMSE total score. It was, however, associated with older age and poorer baseline ADAS-Cog performance. Moreover, at comparable MMSE total scores, patients with greater atrophy performed worse on delayed recall and orientation to time, while performing better on serial sevens. Thus, greater medial temporal atrophy appeared to identify a memory-predominant pattern of impairment rather than simply greater global cognitive severity.

Several mechanisms could account for this association. Severe medial temporal atrophy may reflect more advanced Alzheimer-related neurodegeneration, but it may also indicate coexisting age-related pathology that is not directly addressed by amyloid removal. Limbic-predominant age-related TDP-43 encephalopathy and hippocampal sclerosis are potential examples, particularly in older patients with prominent memory impairment [18]. Neither could be evaluated directly in this study, and the present findings cannot establish the underlying pathology. Nevertheless, amyloid positivity establishes the presence of amyloid pathology and eligibility for anti-amyloid therapy; it does not establish that amyloid is the sole determinant of an individual patient's cognitive impairment or subsequent progression.

This distinction may have practical relevance. Amyloid PET or CSF biomarkers establish amyloid positivity for treatment eligibility, whereas structural MRI is already obtained before treatment for safety screening. The present results suggest that these modalities may provide different types of information: amyloid biomarkers identify the therapeutic target, while structural MRI may provide additional information about the extent of neurodegeneration and subsequent clinical course. Because quantitative medial temporal atrophy is available from routine pretreatment imaging without additional radiation or procedural burden, its potential value for prognostic counselling warrants further investigation.

Baseline cerebral microbleeds provided similarly relevant information about safety. ARIA-H occurred in 6 of 25 patients with at least one baseline microbleed compared with 3 of 136 patients without a microbleed, corresponding to an odds ratio of 14.0. An apparent dose-response relationship was also observed across microbleed categories, and the association persisted after adjustment for age, treatment agent, and white matter hyperintensity burden. In contrast, baseline Centiloid values showed no association with ARIA and were nearly identical in patients who did and did not develop ARIA.

This finding is biologically plausible because cerebral amyloid angiopathy is considered an important substrate of ARIA [19], and cerebral microbleeds are an established imaging marker of vascular amyloid-related injury [20]. Amyloid PET, in contrast, predominantly reflects parenchymal amyloid and does not reliably distinguish the vascular amyloid burden associated with cerebral amyloid angiopathy [21]. Cerebral microbleeds are also more common in East Asian than in Western populations, particularly in deep or infratentorial locations [22], so this marker may be encountered more frequently in Japanese practice than in the trial populations. Baseline microbleed count may therefore provide more relevant pretreatment information about hemorrhagic risk than the Centiloid value. Notably, all patients in this cohort met current treatment eligibility criteria, including four or fewer cerebral microbleeds and no cortical superficial siderosis, yet ARIA-H risk differed substantially according to the presence and number of baseline microbleeds. Risk within the currently eligible range may therefore not be uniform, and baseline microbleed count may provide additional information for pretreatment safety counselling beyond the current binary eligibility threshold. The number of ARIA events in this cohort was limited, however, and these findings require confirmation in larger populations.

The longitudinal lecanemab data provide additional information on cognitive and functional trajectories during routine clinical care. MMSE and CDR-SB worsened progressively over 18 months, and the magnitude of CDR-SB change was broadly comparable with that reported in clinical trials, although patients in the present cohort had less functional impairment at baseline. Decline was not uniform across domains. Memory, orientation, community affairs, and home and hobbies deteriorated progressively, whereas naming, repetition, writing, copying, and immediate recall remained preserved throughout, and personal care was affected only later and to a small degree. These domain-level trajectories may provide useful information when counselling patients and families about the pattern of change that can occur during treatment.

Individual trajectories varied substantially around these group means. This heterogeneity should not be interpreted as variation in individual treatment response because, in the absence of an untreated comparator, observed change cannot be partitioned into underlying disease progression and treatment effect for an individual patient. Nevertheless, the association between pretreatment medial temporal atrophy and subsequent functional decline suggests that at least part of this clinical heterogeneity may be related to characteristics already present before treatment begins.

Donanemab was introduced approximately one year later than lecanemab, and follow-up in this cohort was limited to 6 months. Cognitive and functional measures showed no significant change during this interval, and no individual cognitive or functional domain showed significant worsening. These observations should be regarded as descriptive. Treatment allocation was not randomized, there was no untreated comparator, and the cohorts differed in eligibility criteria, baseline cognitive status, and duration of observation. The observed 6-month stability therefore cannot be attributed to a treatment effect, and direct comparison of effectiveness between the two agents is not appropriate. The lower baseline CDR-SB of this cohort relative to TRAILBLAZER-ALZ 2 is consistent with the absence of tau PET-based enrichment in routine practice, so patients treated in practice may be at an earlier stage than those in whom the agent was tested. Longer follow-up will be required to characterize the real-world cognitive and functional trajectory of patients receiving donanemab.

The absence of measurable cognitive and functional change over 6 months in the donanemab cohort invites one speculative explanation that warrants prospective investigation. Donanemab binds pyroglutamate-modified amyloid β within deposited plaques and therefore acts directly on plaque, producing rapid amyloid removal [23], so cognitive effects might emerge on a different timescale. This interpretation remains speculative: the present observational study did not establish a relationship between early amyloid clearance and cognitive change and cannot demonstrate that the 6-month stability observed in our cohort resulted from treatment. Serial amyloid imaging combined with longer cognitive follow-up will be needed to test this hypothesis.

This study also illustrates several operational challenges associated with delivering anti-amyloid therapy in routine practice. Differences in infusion frequency have direct implications for infusion capacity, while repeated neuropsychological assessments, particularly the time-intensive CDR, place increasing demands on outpatient services as the treated population expands. The CDR additionally requires a knowledgeable informant and therefore could not be obtained in patients who lived alone or attended without an appropriate informant, a practical constraint that is less apparent in clinical trial datasets. Long-term treatment also requires coordination between specialized dementia centers and community facilities. Systematic collection of clinical, safety, and operational data across treatment centers could therefore contribute to national registries and help define how these therapies can be delivered sustainably in routine care.

## 5 Limitations

This study has several limitations. It was retrospective and conducted at a single center, treatment selection was non-randomized, and the lecanemab and donanemab cohorts differed in eligibility criteria, baseline disease stage, and duration of follow-up. The study was therefore not designed to compare the effectiveness or relative safety of the two agents. There was no untreated comparator, and observed cognitive trajectories cannot be attributed to treatment effects. Follow-up for donanemab was limited to 6 months. SWI was not used, and microbleeds assessed on T2*-weighted GRE may have been underestimated [24,25]. CDR could not be obtained in patients who attended without an appropriate informant, a practical limitation of functional assessment in routine clinical care. APOE genotyping was not routinely available, biomarkers of TDP-43 and other non-Alzheimer pathologies were not obtained, and longitudinal ADAS-Cog data were available only in a subset. Power to detect an association between amyloid burden and subsequent change was limited, because only 19 to 31 lecanemab-treated patients had both a Centiloid value and a follow-up assessment at each time point; associations of the magnitude observed (r of about 0.2 to 0.3) could not have been detected. Finally, predictor analyses were exploratory, *P* values are unadjusted, and the findings require validation in larger independent cohorts.

## 6 Conclusions

In this real-world cohort, pretreatment MRI provided prognostic and safety information beyond that provided by amyloid PET burden. Severe medial temporal atrophy was associated with greater subsequent functional decline, while baseline cerebral microbleeds were strongly associated with ARIA-H even within the currently permitted treatment range. Amyloid PET burden was not associated with either outcome. Because structural and vascular MRI information is already available before anti-amyloid therapy, these routinely acquired markers may help refine prognostic and safety counselling without additional testing.

## Funding sources

This study received no specific funding.

## Data availability

The data that support the findings of this study were derived from patient records collected at Tachikawa Hospital and are not publicly available owing to privacy and ethical restrictions. De- identified data may be made available from the corresponding author upon reasonable request with approval from the Tachikawa Hospital Ethics Committee.

## Declaration of generative AI and AI-assisted technologies in the writing process

During the preparation of this work, the corresponding author used Claude (Anthropic) to assist with language editing, manuscript formatting, reference management, verification of the data entered in the analysis spreadsheet against the source records, and checking of internal consistency of numbers and statements throughout the manuscript. After using this tool, the author reviewed and edited the content as needed and takes full responsibility for the content of the publication.

## CRediT authorship contribution statement

**Yuki Kujuro:** Writing – review & editing, Writing – original draft, Visualization, Methodology, Investigation, Formal analysis, Data curation, Conceptualization. **Marina Saito:** Writing – review & editing, Investigation, Data curation. **Manami Maruko:** Writing – review & editing, Investigation, Data curation. **Kazuko Kamiyama:** Resources, Project administration, Investigation. **Aya Nakano:** Resources, Investigation. **Emi Qian:** Investigation. **Teppei Okamura:** Resources, Investigation. **Daisuke Ito:** Writing – review & editing, Supervision, Conceptualization. **Toshimi Kageyama:** Resources, Investigation. **Ichiro Sugiyama:** Investigation. **Eriko Ishihara:** Investigation. **Tatsuro Kuwahara:** Investigation. **Kazunori Moritani:** Resources, Investigation. **Hitoshi Katai:** Writing – review & editing, Supervision. **Hidenori Hattori:** Writing – review & editing, Supervision, Conceptualization.

## Declaration of competing interest

The authors declare the following financial interests/personal relationships which may be considered as potential competing interests: Yuki Kujuro reports a relationship with Tachikawa Hospital that includes: speaking and lecture fees. If there are other authors, they declare that they have no known competing financial interests or personal relationships that could have appeared to influence the work reported in this article.
