# Supplementary material for: Baseline MRI markers and clinical outcomes during anti-amyloid β therapy: A real-world study in a Japanese dementia center
Source: J Prev Alzheimers Dis. 2026 Oct 17;13(10):100686. doi: 10.1016/j.tjpad.2026.100686 (PMC13696334; doi:10.1016/j.tjpad.2026.100686)
Supplement: Supplementary file 1 [file mmc1.docx]

**Supplementary Material**

Baseline MRI Markers and Clinical Outcomes During Anti-Amyloid β Therapy: A Real-World Study in a Japanese Dementia Center

**Supplementary Table S1. Longitudinal Changes in Cognitive Outcomes**

| **Drug** | **Outcome** | **Time point** | **No.** | **Mean (SD)** | **No. paired** | **Mean change (SD)** | **P value** |
| --- | --- | --- | --- | --- | --- | --- | --- |
| Lecanemab | MMSE | Baseline | 87 | 24.95 (2.24) | — | — | — |
|  |  | 6 months | 71 | 24.00 (2.71) | 70 | -0.80 (2.59) | .012 |
|  |  | 12 months | 56 | 23.02 (3.02) | 55 | -1.78 (3.22) | <.001 |
|  |  | 18 months | 32 | 22.62 (2.79) | 31 | -2.26 (2.68) | <.001 |
|  | CDR-SB | Baseline | 69 | 2.17 (0.95) | — | — | — |
|  |  | 6 months | 58 | 3.02 (1.52) | 58 | +0.79 (1.14) | <.001 |
|  |  | 12 months | 44 | 3.38 (1.85) | 44 | +1.10 (1.52) | <.001 |
|  |  | 18 months | 29 | 3.83 (2.07) | 29 | +1.60 (1.68) | <.001 |
|  | ADAS-Cog | Baseline | 76 | 13.00 (4.79) | — | — | — |
|  |  | 6 months | 29 | 13.10 (4.57) | 27 | +0.49 (4.81) | .599 |
|  |  | 12 months | 23 | 15.70 (5.04) | 22 | +2.89 (5.33) | .019 |
|  |  | 18 months | 9 | 15.42 (4.25) | 8 | +1.08 (2.52) | .266 |
| Donanemab | MMSE | Baseline | 72 | 23.75 (2.67) | — | — | — |
|  |  | 6 months | 26 | 23.54 (3.18) | 26 | +0.08 (2.95) | .895 |
|  | CDR-SB | Baseline | 53 | 2.73 (1.41) | — | — | — |
|  |  | 6 months | 23 | 2.87 (1.30) | 22 | +0.23 (1.05) | .324 |
|  | ADAS-Cog | Baseline | 56 | 14.33 (4.67) | — | — | — |
|  |  | 6 months | 10 | 14.96 (6.36) | 10 | +1.08 (3.18) | .311 |

Scores are shown as mean (SD) among all patients assessed at that time point. Change from baseline was calculated as a paired difference (Δ = follow-up − baseline) among patients with both assessments, and the number of paired observations is given separately. P values were calculated with paired t tests. Negative Δ indicates worsening for MMSE; positive Δ indicates worsening for CDR-SB and ADAS-Cog.

Abbreviations: ADAS-Cog, Alzheimer Disease Assessment Scale–Cognitive Subscale; CDR-SB, Clinical Dementia Rating–Sum of Boxes; MMSE, Mini-Mental State Examination; SD, standard deviation.

**Supplementary Table S2. All ARIA Events in Donanemab-Treated Patients**

| **Patient** | **ARIA type** | **Infusion No.** | **Dosing regimen** | **Premedication** | **Symptomatic** | **Treatment discontinued** |
| --- | --- | --- | --- | --- | --- | --- |
| 1 | ARIA-E | 2 | Original regimen | No | No | No |
| 2 | ARIA-E | 2 | Original regimen | Yes | No | No |
| 3 | ARIA-E + ARIA-H | 3 | Modified titration | Yes | No | No |
| 4 | ARIA-E + ARIA-H | 6 | Original regimen | No | No | Yes |
| 5 | ARIA-H | 1 | Original regimen | No | No | No |
| 6 | ARIA-H | 2 | Modified titration | Yes | No | No |

ARIA events were detected on scheduled magnetic resonance imaging surveillance and were clinically asymptomatic in all cases. The dosing regimen is categorized as the original regimen or the modified titration regimen implemented in Japan during the study period following the TRAILBLAZER-ALZ 6 study of donanemab.

Abbreviations: ARIA, amyloid-related imaging abnormality; ARIA-E, amyloid-related imaging abnormality with edema or effusion; ARIA-H, amyloid-related imaging abnormality with hemorrhage or hemosiderin.

**Supplementary Table S3. All Infusion-Related Reaction Events in Donanemab-Treated Patients**

| **Patient** | **Infusion No.** | **Premedication** | **Dosing regimen** | **Clinical manifestations** | **Treatment discontinued** |
| --- | --- | --- | --- | --- | --- |
| 1 | 2 | No | Original regimen | Warm sensation, dyspnea | Yes |
| 2 | 2 | Yes | Original regimen | Dyspnea | No |
| 3 | 2 | Yes | Modified titration | Chest pain | No |
| 4 | 4 | No | Original regimen | Nausea, chills | Yes |
| 5 | 4 | No | Original regimen | Dyspnea, flushing | Yes |
| 6 | 4, 9 | Yes (4th and 9th infusions) | Modified titration | 4th: throat discomfort; 9th: dyspnea | Yes |
| 7 | 5, 6 | Yes (6th infusion) | Modified titration | 5th: chills, flushing; 6th: hypotension | Pending |
| 8 | 5 | No | Modified titration | Chills | No |

Infusion numbers correspond to the treatment cycle at which the reaction occurred.

Abbreviations: IRR, infusion-related reaction.

**Supplementary Figure S1. Domain-Level Change in MMSE and CDR Subdomains**

Mean change from baseline with 95% confidence intervals for each subdomain, shown for donanemab at 6 months and for lecanemab at 6, 12, and 18 months. Positive values indicate improvement (higher MMSE and lower CDR subdomain scores). Red denotes subdomains with significant worsening and blue significant improvement, defined as a 95% confidence interval excluding zero; grey denotes intervals that include zero. The horizontal axis scale is unified across time points within each instrument but differs between instruments because the two scales have different ranges.

Abbreviations: CDR, Clinical Dementia Rating; CI, confidence interval; MMSE, Mini-Mental State Examination.

**Supplementary Figure S2. Kaplan-Meier Estimates of Treatment Persistence**

Discontinuation was defined as cessation for any cause. Vertical ticks indicate censoring. Curves are truncated where fewer than 5 patients remain at risk. Numbers at risk are shown beneath the curves. For lecanemab, discontinuations occurring at the 18-month evaluation milestone were included as events but were excluded when summarizing reasons for discontinuation.
